# All-inside anterior cruciate ligament reconstruction with and without anterolateral ligament reconstruction: a prospective study

**DOI:** 10.1186/s12891-023-07128-9

**Published:** 2024-01-02

**Authors:** Ali Torkaman, Mehdi Hosseinzadeh, Elham Mohammadyahya, Parsa Torkaman, Mohammad Reza Bahaeddini, Amir Aminian, Hamed Tayyebi

**Affiliations:** 1 Department of Orthopedics, School of Medicine, Iran University of Medical Sciences, Tehran, Iran; 2 Bone and Joint Reconstruction Research Center, Shafa Orthopedic Hospital, Shafa Yahyaian Educational and Medical Center, Iran University of Medical Science, Baharestan Square, Mojahedin-e-Islam St, Tehran, 1157637131 Iran

**Keywords:** Anterior cruciate ligament, Anterior cruciate ligament reconstruction, Anterolateral

## Abstract

**Background:**

There is no clear consensus regarding the superiority of a combined anterior cruciate ligament reconstruction (ACLR) with anterolateral ligament reconstruction (ALLR) versus an isolated ACLR. In this study, we compared the postoperative stability profile, complications, and patient-reported outcomes of these procedures.

**Methods:**

Twenty-one patients with an anterior cruciate ligament (ACL) tear who were either treated by an isolated all-inside ACLR (n = 21) or a combined all-inside ACLR and ALLR (n = 20) were included. The outcomes were evaluated in the last follow-up and included the postoperative stability profile evaluated by the Lachman test, pivot shift test, and KT-1000 side-to-side difference, postoperative complications, and patient-reported outcomes evaluated by the International Knee Documentation Committee (IKDC) score and Lysholm knee scale.

**Results:**

The baseline characteristics of the two groups were not significantly different. The residual Lachman and pivot shift were not significantly different between the two groups (P = 0.41 and P = 0.18, respectively). The mean KT-1000 side-to-side difference was 1.93 ± 1.9 mm in the isolated and 1.635 ± 0.91 mm in the combined group (P = 0.01). The mean improvement of the IKDC score was not significantly different between the isolated and combined groups (24.7 vs. 25.2, P = 0.28). The mean improvement of the Lysholm scale was not significantly different between the isolated and combined groups (33.5 vs. 34.1, P = 0.19). ACL re-rupture occurred in three patients of the isolated group and no patient of the combined group.

**Conclusion:**

The outcomes of patients in the present study support performing a combined ALL and ACL reconstruction.

## Introduction

Anterior cruciate ligament (ACL) tears are among the most common knee injuries. The incidence of ACL tears has steadily increased over the last decades, partly because of more women’s involvement in sports activities [1]. The number of ACL reconstruction (ACLR) surgeries has also increased in parallel. Although ACLR successfully restores knee stability and function in the majority of patients, knee rotational stability is not completely restored in a considerable number of cases [2].

Persistent rotational instability after ACLR is associated with worse postoperative outcomes [3]. Anterolateral ligament (ALL) injury has been acknowledged as an underlying potential component in post-ACLR rotational instability [4]. ALL is an anterolateral stabilizer of the knee, which plays an important role in the tibial rotational control at various degrees of knee flexion, thereby helping the overall knee stability [5]. For this reason, combined ACLR and ALL reconstruction (ALLR) has been suggested as an option to reduce the rate of post-ACLR rotational instability [6].

Despite numerous comparative studies of the outcomes and several meta-analysis studies published in this regard, there is still no consensus about the reliability of combined ACL and ALL reconstruction, and further studies are required to determine the efficacy of combined procedures in the clinical setting [7].

In this study, we aimed to compare the effect of an isolated standard ACLR versus a combined ACLR and ALLR procedure on postoperative stability, complications, and clinical outcomes of patients with ACL rupture. We hypothesized that combined ACLR and ALLR provides superior outcomes to ACLR alone.

## Methods

This study was approved by the review board of our institute under the code IR.IUMS.FMD.REC.1398.270. Patients provided written informed consent before participation in the study. Between 2016 and 2021, patients with an ACL tear who were referred to the orthopedic clinic of our university hospital and underwent ACLR with or without ALLR were prospectively evaluated. The inclusion criteria were unilateral ACL injury, use of quadruple-bundle Semitendinosus- tendon autograft for ACLR, intact meniscus, a pivot shift test of 2 + or 3+, and a minimum follow-up of two years. Patients with multiple trauma or multi-ligament injuries, revision ACLR, patients with injuries causing neurological deficits, patients with a history of injury or surgery in the ipsilateral or the contralateral knee, patients with disorders affecting the knee joints and muscles such as rheumatoid arthritis, and patients who were lost to follow-up were excluded from the study. Forty-one patients who met the study criteria were included in the final analysis.

### Surgical Procedures

All the surgeries were done by one senior knee surgeon who used to perform isolated ACLR before 2019 and a combined ACLR and ALLR procedure afterward. ACLR was performed using a standard all-inside technique in both groups. A four-strand semitendinosus tendon autograft was used for the ACLR, which was fixed with an adjustable endobutton (Arthrex Naples, FL, USA) at both ends. Gracilis tendon was used for ALLR. For this purpose, we first created two tunnels, one of which was located 5 mm posterior and 5 mm proximal to the lateral epicondyle of the femur and the other 1 cm below the joint line between the head of the fibula and the Gerdy’s tubercle. Then, we passed the graft beneath the iliotibial band and through the two tunnels. Finally, we fixed the graft with the help of a bioscrew (Arthrex Naples, FL, USA).

### Postoperative rehabilitation

Knee range of motion (ROM) was started immediately after the operation. Quadriceps strengthening and hamstring stretch exercises were started the day after the operation to achieve full active knee extension and 90º of flexion within two weeks. Weight-bearing, as tolerated, was advised at the same time. Ambulation without crutches or canes was advised after the patients achieved normal gait. Physiotherapy was implemented as soon as possible to improve muscle strength, proprioception, and range of motion. Jogging, running, and household activities were advised at 4 to 5 months, and return to sports was allowed after 12 months.

### Outcome measures

Demographic characteristics of the patients were collected prospectively. Outcome measures were evaluated before the operation and in the last follow-up visit. Knee stability was assessed using the Lachman and pivot shift tests. The Lachman test was categorized based on the amount of maximum anterior tibial translation into four grades, including 0 (< 3 mm), 1+ (3–5 mm), 2+ (6–10 mm), and 3+ (> 10 mm). The pivot shift test was also categorized into four grades, including 0 (negative), 1+ (glide), 2+ (clunk), or 3+ (gross). To measure the side-to-side difference of the anterior tibial translation a KT-1000 arthrometer (MEDmetric^®^ Corp., San Diego, CA, USA) was used at 30º of knee flexion with a force of 30 pounds (133 Newtons) [8]. The knee function was evaluated using the Lysholm scale and the International Knee Documentation Committee (IKDC) subjective knee scale. Based on the Lysholm scale, a score between 0 and 100 was assigned to each patient, and a higher score was representative of better knee function. The knee function was also categorized into excellent (score 91–100), good (score 84–90), fair (score 65–83), and poor (score < 64) [9]. IKDC was evaluated using the Persian translated form [10, 11], and each patient received a score within the range of 0 and 100, corresponding to the lowest and highest knee function, respectively.

### Sample size and statistical analysis

The sample size was determined according to the mean and standard deviation (SD) of the Lysholm scale provided in the study of Saithna et al. (95.4 ± 5.3 vs. 90.0 ± 7.1) [12]. Using this data, a type I error of 0.05 and a power of 80%, a number of 20 patients in each study group was found to be enough to detect a significant difference using an independent t-test.

Statistical analyses were done using the SPSS for Windows, version 16 (SPSS Inc., Chicago, Ill., USA). Descriptive data were presented by mean ± SD or number (percentages). The mean value of quantitative variables in the two study groups was compared with an independent t-test or its nonparametric equivalent (Mann–Whitney U test) in case of non-normal distribution. Qualitative variables were compared using a chi-squared or Fisher’s exact test. P < 0.05 was considered significant.

## Results

Twenty-one patients who were treated by isolated ACLR and 20 patients who were treated by combined ACL and ALL reconstruction were included in the analysis. The baseline characteristics of the patients were not significantly different between the two study groups (Table 1).

**Table 1:** Comparison of baseline characteristics between the two study groups

Variable	Isolated ACLR (n=21)	Combined ACLR & ALLR (n = 20)	P-value
Age (year)	26.7 ± 8.9	25.9 ± 6.9	0.21
BMI (kg/m^2^)	24.5 ± 2.2	24.2 ± 2.1	0.56
**Sex**			
• Male • Female	17 (81) 4 (19)	18 (85.7) 2 (14.3)	0.64
**Laterality**			
• Right • Left	10 (47.6) 11 (52.4)	9 (45) 11 (55)	0.78
**Preoperative Lachman test**			
• 2+ • 3+	8 (38.1) 13 (61.9)	8 (40) 12 (60)	0.66
**Preoperative pivot shift test**			
• 2+ • 3+	8 (38.1) 13 (61.9)	8 (40) 12 (60)	0.66
Follow-up (months)	39.8 ± 14.1	41.3 ± 15.5	0.42

ACLR: Anterior cruciate ligament reconstruction; ALLR: Anterolateral ligament reconstruction; BMI: Body mass index. Data are presented as mean ± SD or number (%). P < 0.05 is considered significant

At the last follow-up visit, the Lachman test was positive in five (23.8%) patients of the isolated group and two (10%) patients of the combined group. This difference was not statistically significant (P = 0.41). The pivot shift test was also positive in five (23.8%) patients of the isolated group and one (5%) patients of the combined group. This difference was not statistically significant (P = 0.18). The mean KT-1000 side-to-side difference was 1.93 ± 1.9 mm in the isolated and 1.65 ± 0.91 mm in the combined group. This difference was statistically significant (P = 0.01).

The mean IKDC score of the isolated ACLR group was 54.3 ± 8.6 before the operation and 79 ± 9.2 at the last follow-up. This difference was statistically significant (P < 0.001). The mean IKDC score of the combined ACLR and ALLR group was 56.3 ± 8.8 before the operation and 81.5 at the last follow-up. This difference was statistically significant (P < 0.001). The mean improvement of the IKDC score was not significantly different between the isolated and combined groups (24.7 vs. 25.2, P = 0.28).

In the isolated ACLR group, the mean Lyshom scale improved from a mean value of 55.6 ± 9.5 before the operation to a mean value of 89.1 ± 8.7 at the last follow-up (P < 0.001). Accordingly, the outcome was good to excellent in 15 of 21 patients (71.4%). In the combined ACLR and ALLR group, the mean Lyshom scale improved from a mean value of 56.1 ± 9.8 before the operation to a mean value of 90.2 ± 9.3 at the last follow-up (P < 0.001). Accordingly, the outcome was good to excellent in 19 of 20 patients (95%). The mean improvement of the Lysholm scale was not significantly different between the isolated and combined groups (33.5 vs. 34.1, P = 0.19). The outcome measures of the two study groups are demonstrated in detail in Table 2.

**Table 2:** Comparison of outcome measure between the two study groups

Variable	Isolated ACLR (n=21	Combined ACLR & ALLR (n = 20)	P-value
**Postoperative Lachman test**			
• 0 • 1+ • 2+	16(76.2%) 1(4.8%) 4(19%)	18(90%) 1(5%) 1(5%)	0.41
**Postoperative pivot shift test**			
• 0 • 1+ • 2+	16(76.2%) 3(14.3%) 2(9.5%)	19(95%) 0 1(5%)	0.18
KT1000 side-to-side difference (mm)	1.93 ± 1.9	1.65 ± 0.91	0.01
IKDC score improvement	24.7 ± 7.2	25.2 ± 6.8	0.28
Lysholm scale improvement	33.5 ± 8.1	34.1 ± 6.9	0.19
**Outcome according to the Lysholm scale**			
• Excellent • Good • Fair • Poor	13(76.2%) 2(9.5%) 3(14.3%) 3	16(80%) 3(15%) 1(5%) 0	0.72

ACLR: Anterior cruciate ligament reconstruction; ALLR: Anterolateral ligament reconstruction; IKDC: International Knee Documentation Committee. Data are presented as mean ± SD or number (%). P < 0.05 is considered significant

### Postoperative Complications

One patient in each group had postoperative arthrofibrosis that reached full ROM within six months without the need for extra intervention. Eight (38%) patients in the isolated ACLR group and seven (35%) patients in the combined group had anterior knee pain (P = 0.35). Three patients in the isolated group experienced an ACL re-rupture, two of which occurred during soccer and one of which during ping pong playing. Two of these patients also had medial Bucket Handle meniscus tears. None of these patients had increased posterior tibial slope or contralateral rupture. Two of these patients had generalized ligamentous laxity. Despite generalized ligamentous laxity in three patients of the combined group, no patients in the combined group experienced a re-rupture. No other postoperative complications were observed.

## Discussion

In this study, we compared the postoperative stability, complications, and clinical outcomes of the patients with an ACL tear who were managed with isolated all-inside ACLR or all-inside ACLR combined with ALLR. According to our investigation, the postoperative Lachman test and pivot shift test were not significantly different between the two study groups. However, the KT-1000 side-to-side difference was significantly smaller in the combined group. Also, the rate of postoperative complications, including ACLR failure, was higher in the isolated group (14.3% vs. 0%). The knee function evaluated by the IKDC score and Lysholm scale was not significantly different between the isolated and combined groups.

The outcomes of isolated ACLR and combined ACLR and ALLR have been studied in numerous studies. Several systematic and review meta-analyses have also been published in this regard.

Na et al., in a systematic review with meta-analysis, compared the efficacy of ACLR with and without anterolateral extra-articular procedures (AEAPs), including anterolateral ligament reconstruction (ALLR) or lateral extra-articular tenodesis (LET). Twenty studies, including 11 randomized controlled trials, were included in their analysis. Based on their results, the combined group had superior pivot-shift grades and graft failure rates compared to the isolated group, regardless of the AEAP technique. Also, the improvement of subjective function was slightly higher in the combined group. ALLR seemed to be a better extra-articular procedure for improving rotational stability when compared with LET. Accordingly, they suggested performing a combined ACLR and ALLR for the treatment of ACL tears [13].

Rhatomy et al., in a systematic review and meta-analysis, compared the clinical outcomes and rotational stability of ACLR with or without ALLR. Six studies that met the meta-analysis criteria were included. The combined group tended to have superior laxity outcomes. The clinical outcomes were also superior in the combined group, particularly in the absence of residual laxity. However, other results, such as graft failure, were not significantly different between the two groups [14].

Beckers et al. performed a systematic review and meta-analysis to determine if a combined lateral augmentation and ACLR provide better outcomes compared to an isolated ACLR. Eleven studies (1892 patients) were included in their analysis. Lateral augmentation was ALLR in six studies, and different types of Lateral Extra-articular Tenodesis (LET) in the remaining. Patients who underwent lateral augmentation had a significantly lower rate of graft failure (3% vs. 12%). Moreover, rotational laxity was significantly lower in patients who underwent lateral augmentation (6% vs. 14%). The addition of lateral augmentation also reduced anterior tibial translation. The patient-reported outcomes, including IKDC and Tegner, were not significantly different between the two groups, while the Lysholm score was in favor of the combined procedure [15].

Delaloye et al. reviewed available studies reporting on clinical outcomes after isolated or combined ACLR and ALLR. Only studies with a minimum follow-up of two years were included (n = 5). The rate of graft failure rate was 2.5 times lower in the combined group when bone patella tendon-bone graft was used and 3.1 times lower when hamstring graft was used for ACLR. Also, failure of the medial meniscal repair was two times lower in the combined group. However, functional outcomes and return to sport did not reveal any significant difference between the two groups. Combined ACLR and ALLR were not associated with an increased rate of reoperation [16].

Saithna et al., in a narrative review study, compared the outcomes of isolated ACLR and combined ACLR and ALLR. According to their review, the combined procedure was associated with improved outcomes, including a significantly lower risk of graft failure, a significantly lower risk of reoperation for secondary meniscectomy, a significantly increased rate of return to the preinjury sport level, less residual pivot shift, and superior IKDC (92.7 vs. 87.1) and Lysholm scores (95.4 vs. 90) [12].

Lima et al. also compared the clinical outcomes of isolated ACLR with combined ACLR and ALLR in a systematic review and meta‑analysis study. Studies that did not use “anatomical” techniques for ALLR and studies with follow-ups of fewer than two years were excluded. Ten studies that met the study criteria were analyzed, including 674 patients in the isolated ACLR group and 821 patients in the combined ACLR and ALLR group. The combined group was revealed to have superior pivot shift, re-rupture rate, Lachman test, and Lysholm score [17].

Reviewing the literature reveals that there are still some areas of controversy about the superiority of combined ACLR and ALLR versus isolated ACLR. While some studies show positive effects of the combined procedure on patient-reported outcomes, the other studies report no significant difference in this regard. However, the majority of studies report at least one advantage of the combined procedure when compared with the isolated ACLR, particularly the lower rate of failure. Similar to the earlier studies, we observed some evidence in favor of doing a simultaneous ALL reconstruction, particularly the significantly lower KT-1000 side-to-side difference and a lower rate of re-rupture in this group.

In addition, the differences in stability measures (Lachman and pivot shift test), although not statistically significant, tend in favor of the combined all-inside ACLR and ALLR procedure.

It is worth noticing that we used all-inside ACLR for all patients in the present study. In the all-inside procedure, fixation of the graft with an adjustable endobutton at both ends allows for the use of a shorter graft, which instead provides the opportunity to create a four-stranded semitendinosus graft with greater stability. In addition, the gracilis tendon will be completely left for ALLR, making a more stable ALLR. However, ACLR procedures other than all-inside require a longer length of graft. For this reason, part of the gracilis tendon will also be used in ACLR. Even so, the final prepared graft is still thinner than the graft used in the all-inside technique. Moreover, the remnant of gracilis will be used for ALLR, as its shorter length and diameter cause smaller ALLR stability. As a result, the all-inside technique could provide better graft stability for both ACLR and ALLR compared to the other ACLR procedures.

The present study was not without limitations. The main limitation of the study was the small number of patients that could have adversely affected the power of the study. For example, the pivot shift test was positive in three (23.8%) patients of the isolated group and one (5%) patient of the combined group. This difference, which was not significant in this paper, could be significant with a higher number of patients. Therefore, the presented results should be confirmed in future complementary studies with larger patient numbers.

## Conclusion

Compared to an isolated all-inside ACLR, a combined all-inside ACLR and ALLR procedure is associated with a lower rate of re-rupture and a reduced KT-1000 side-to-side difference. Also, the knee stability profile does tend to be superior in the combined group. Even so, these results should be interpreted in light of study limitations, particularly the small number of patients.

## Data Availability

The datasets used and/or analysed during the current study are available from the corresponding author on reasonable request.
